# Plant protection product dose rate estimation in apple orchards using a fuzzy logic system

**DOI:** 10.1371/journal.pone.0214315

**Published:** 2019-04-24

**Authors:** Peter Berk, Denis Stajnko, Marko Hočevar, Aleš Malneršič, Viktor Jejčič, Aleš Belšak

**Affiliations:** 1 University of Maribor, Faculty of Agriculture and Life Sciences, Chair of Biosystem Engineering, Maribor, Slovenia; 2 University of Ljubljana, Faculty of Mechanical Engineering, Laboratory for hydraulic machines LVTS, Ljubljana, Slovenia; 3 Agricultural Institute of Slovenia, Department for Agriculture Engineering, Ljubljana, Slovenia; 4 University of Maribor, Faculty of Mechanical Engineering, Laboratory for Structure Evaluation, Maribor, Slovenia; Universiti Sains Malaysia, MALAYSIA

## Abstract

In the process of applying a plant protection product mixed with water (spray mixture) at the prescribed concentration with conventional sprayers for chemical protection of tree canopies in an orchard, standard models are used to express the dose rate of the plant protection product. Characteristic properties of the tree canopy in an orchard are not taken into consideration. Such models result in fixed quantities of spray mixture being sprayed through individual nozzles into a tree canopy. In this research work, an autonomous system is presented, which ensures a controlled quantity of spray mixture sprayed through the nozzles onto different tree canopy segments. The autonomous system is based on a fuzzy logic system (FLS) that includes information about the estimated leaf area to ensure more appropriate control of the spray mixture. An integral part of the FLS is a fuzzy logic controller for three electromagnetic valves operating in the pulse width mode and installed on the axial sprayer prototype. The results showed that, with the FLS, it was possible to control the quantity of spray mixture in the specific range depending on the estimated value of the leaf area, with a quantitative spray mixture average saving of 17.92%. For the phenological growth stage BBCH 91, this method represents a powerful tool for reducing the quantity of spray mixture for plant protection in the future.

## Introduction

Plants can be protected in different ways, chemical protection with plant protection products (PPP) mixed with water, i.e. spray mixture, being the most important one. The purpose of using this method is to destroy harmful organisms and to prevent infection of plants; however, only part of the spray mixture remains on the plant, while part of it goes into the surrounding environment. The remains of the spray mixture lead to pollution of the land, groundwater, air, plants and animals, which represents a serious issue in modern agriculture, more specifically in fruit production. For stable and sustainable fruit production, effects harmful to the environment we live in will have to be reduced. Smaller spray mixture quantities will have to be applied; however, the quality of crop protection in an orchard will have to be retained. This can be ensured with a selective and precise spray mixture application process using an advanced measurement and control system, whereby the process of determining the spray mixture dose rate through the nozzles will be controlled on the basis of an intelligent fuzzy logic system (hereinafter FLS).

### Empirical dose rate determination in permanent crops

For dose rate calculation in permanent crops, fruit growers use a range of empirical models (the per hectare model, TRV model, per leaf surface wall model, etc.) recommended in a series of recent publications [1–6]. These models enable a fixed calculation of the quantities of water and PPP in an orchard. Four decades ago, investigators [2] and [7] established that it would be sensible to consider the characteristic properties of tree canopies in the application process and that it is not suitable to apply the same dose to both small and large tree canopies in orchards, without taking into consideration the total leaf surface in a tree canopy.

### Decision-making models for spray mixture dose rate application in permanent crops

There are many different deciosion-making dose rate control models for permanent crops. In the beginning, the first such models were based on the principle of simple algorithms (decision-making models) that allowed spray mixture control in the ON/OFF mode. Later, owing to the simple decision-making logic of dose rate control, models began to be developed that allowed dose rate control in a discrete way. In recent decades, models enabling continuous dose rate control have begun to be developed.

The ON/OFF control mode is based on Boolean logic, with two possible values: 0 and 1. Dose rate control is based on the information about the presence of the tree canopy that is included in the decision-making model; on the basis of this, the decision-making model enables dose rate control. Investigators [8] were the first to describe the simple decision-making model including an ON/OFF control mode and an optical measurement system to detect tree canopies. Investigator [9] was the first to develop a commercially accessible patented sprayer prototype. Investigator [9] placed an ultrasound measurement system on the sprayer prototype to enable the detection of tree canopies in an orchard and, based on the measured details about the distance and using the ON/OFF mode, he activated the dose rate application process through the nozzles at five different heights. As pointed out in [10] describe the ON/OFF dose rate control model based on the Relative Load Factor (RLF). The distance measured by using ultrasound measurement components at three different heights (at the top, in the middle and in the lower part of a tree canopy) was taken into consideration for calculating the RLF [10]. In recent publication [11] developed an ON/OFF dose rate control mode that enabled control only in case of moving the sprayer and simultaneous detection of a tree canopy at a distance interval measured with an ultrasound measurement system. A similar system was used by [12], except that they used optical sensors to detect tree canopies in an orchard. For dose rate control, investigators [13] used a decision-making model based on the detection of the leaf surface density with a dedicated ultrasound measurement instrument. In recent publication [14] developed a decision-making model based on Lukasiewitz logic and enabling dose rate control using the following three discrete values: no dose, minimal dose and maximum dose. Investigators [15] established that, in order to ensure uniform dose rate application and reduced loss of spray mixture in the surroundings, a continuous real-time control system should be used and determined the maximum spray mixture dose rate depending on the largest measured tree canopy width in an orchard and the minimum value when a tree canopy was absent. For continuous dose rate control, used a control module to generate pulse-width signals based on which the dose rate application was controlled between 0% and 100% [16]. In recent publication [17] placed a LIDAR measurement system on the sprayer prototype. With the LIDAR measurement system, they enabled measurement of the tree canopy volume in an orchard. Information about the measured tree canopy volume was included in the decision-making model. Via a proportionate control system, the decision-making model enabled continuous spray mixture dose rate control. As one of the dose rate reduction possibilities in a recent publications [18, 19] recommend the decision-making model for continuous dose rate control in orchards, based on the ratio of tree canopy height to row spacing.

Fruit growers’ wish to be able to apply spray mixture drops at higher travel speeds of a sprayer is growing; this would shorten the spray mixture application process. Additionally, dose rate control could be ensured with electronic control systems that work on intelligent decision-making models. Certain deficiencies can be detected in relation to all the above decision-making models. Dose rate control in the ON/OFF mode is the simplest; the decision-making models open or close the EMV through which the spray mixture is expressed, based on information about the presence or absence of a tree canopy at the selected distance interval. Investigators [14] enhanced the dose rate control, using a decision-making model that enables dose rate control based on three discrete values. However, it must be stressed that the model is still based on the principle of simple detection of presence or absence of tree canopies. Only after the development of the LIDAR measurement technology did investigators [17] enable measurement of the tree canopy volume, on the basis of which a more precise dose rate control through proporotionate EMV became possible.

In recent years investigators [20] have proposed a real-time method based on an array of ultrasonic sensors to estimate the canopy density in apple orchards and vineyards. Such estimation is used as a reference for adjusting the spraying machine parameters according to the canopy in order to improve droplet deposition on leaves while avoiding drift. In the research work [21], the authors described the application of the Kinect system in the area of precision spray control. For dose-response studies in Plant Protection, logarithmic sprayers are used for a wide range of pesticides [22]. Investigators [23] presented a novel, autonomous, air-assisted sprayer, which is designed with a genetic algorithm to provide automatic spraying and to improve the uniformity of droplet deposition. However, it must be stressed that each of the above-mentioned continuous or other decision-making models is based on a strictly determined mathematical model.

As shown in a series of recent publications [24–31], to develop a genuinely useful statistical predictor for a statistical system, one should observe the guidelines of Chou’s 5-step rule [32] and proceed deliberately through the following five guidelines: (i) how to construct or select a valid benchmark dataset to train and test the predictor; (ii) how to formulate the statistical samples with an effective mathematical expression that can truly reflect their intrinsic correlation with the target to be predicted; (iii) how to introduce or develop a powerful algorithm (or engine) to operate the prediction; (iv) how to properly perform cross-validation tests to objectively evaluate the anticipated accuracy of the predictor; (v) how to establish a user-friendly web-server for the predictor that is accessible to the public.

## Materials and methods

### Using a FLS to determine dose rate in permanent crops

In the control technique, complex dynamic systems with non-linear behaviour or behaviour changing over time are often present; thus, it is often difficult to select variables and characteristics of model parameters. The fuzzy model had been used by many previous investigators (see, e.g., [33–39]).

Based on fuzzy control models, human experience (of fruit growers and phytopathologists) can be used and included in the dose rate control process, as pointed in a recent publications (see, e.g., [40–41]. Thus, control problems can be solved without determining a precise mathematical model, which is required by the classic control approach. If compared to the classic approach, in the dose rate control process, fuzzy models can include an optional number of process variables (measured leaf area and volume, sprayer travel speed, chemical, phytopharmaceutical and biological properties of the functioning of the spray mixture, experience of phythopathologists and fruit growers, orchard age and type, other orchard-specific properties, etc.), and it is possible to enhance the dynamic properties of the control process for spray mixture application. In the literature available to us, no contributions by other authors were found using FLS for PPP application in permanent crops.

### Experiment design

**Fig 1:**
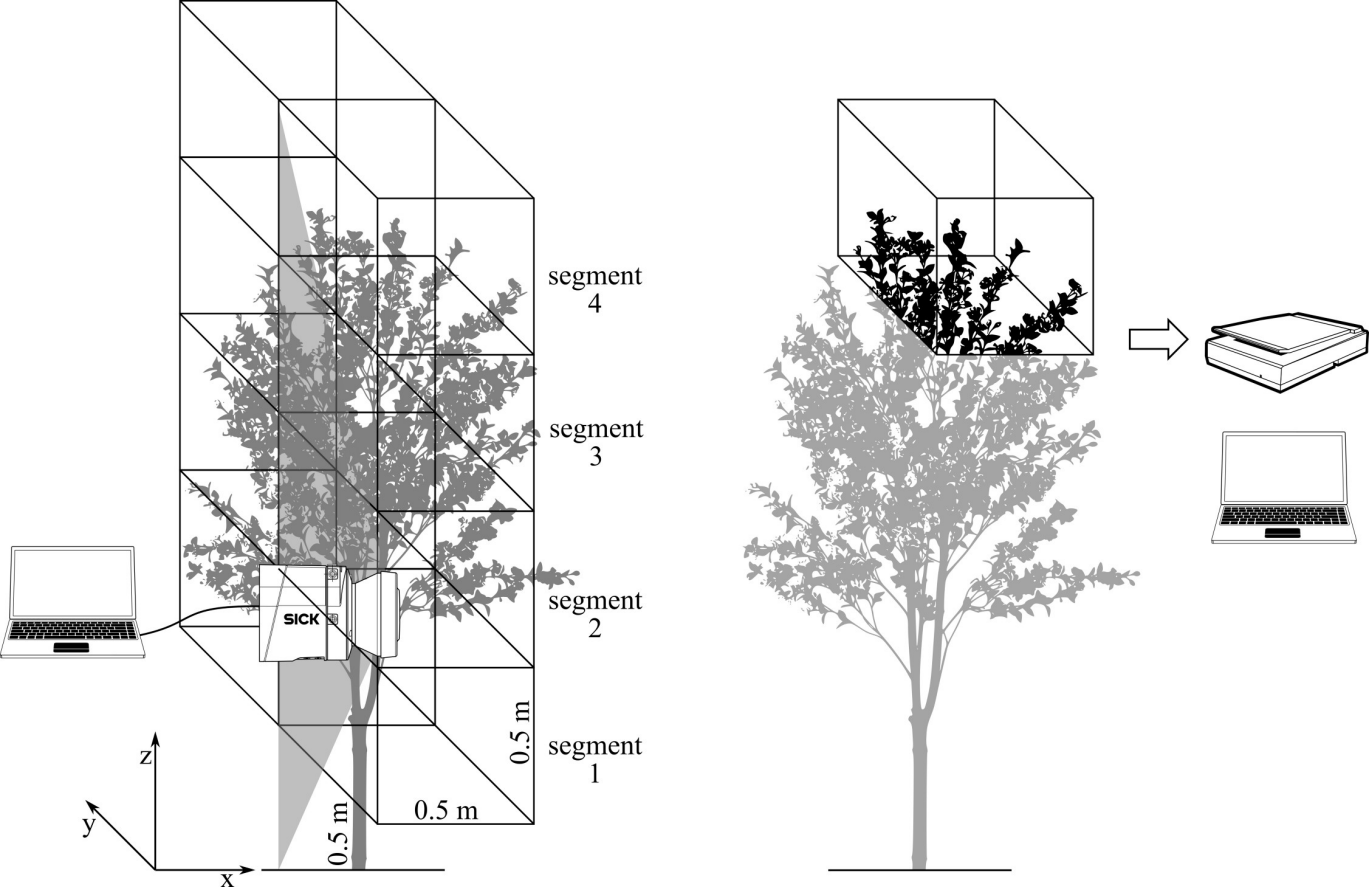
Reconstruction of the tree crown on the principle of measurement with the LIDAR measuring system (left) and manual measurement of the leaf area (right).

**Fig 2:**
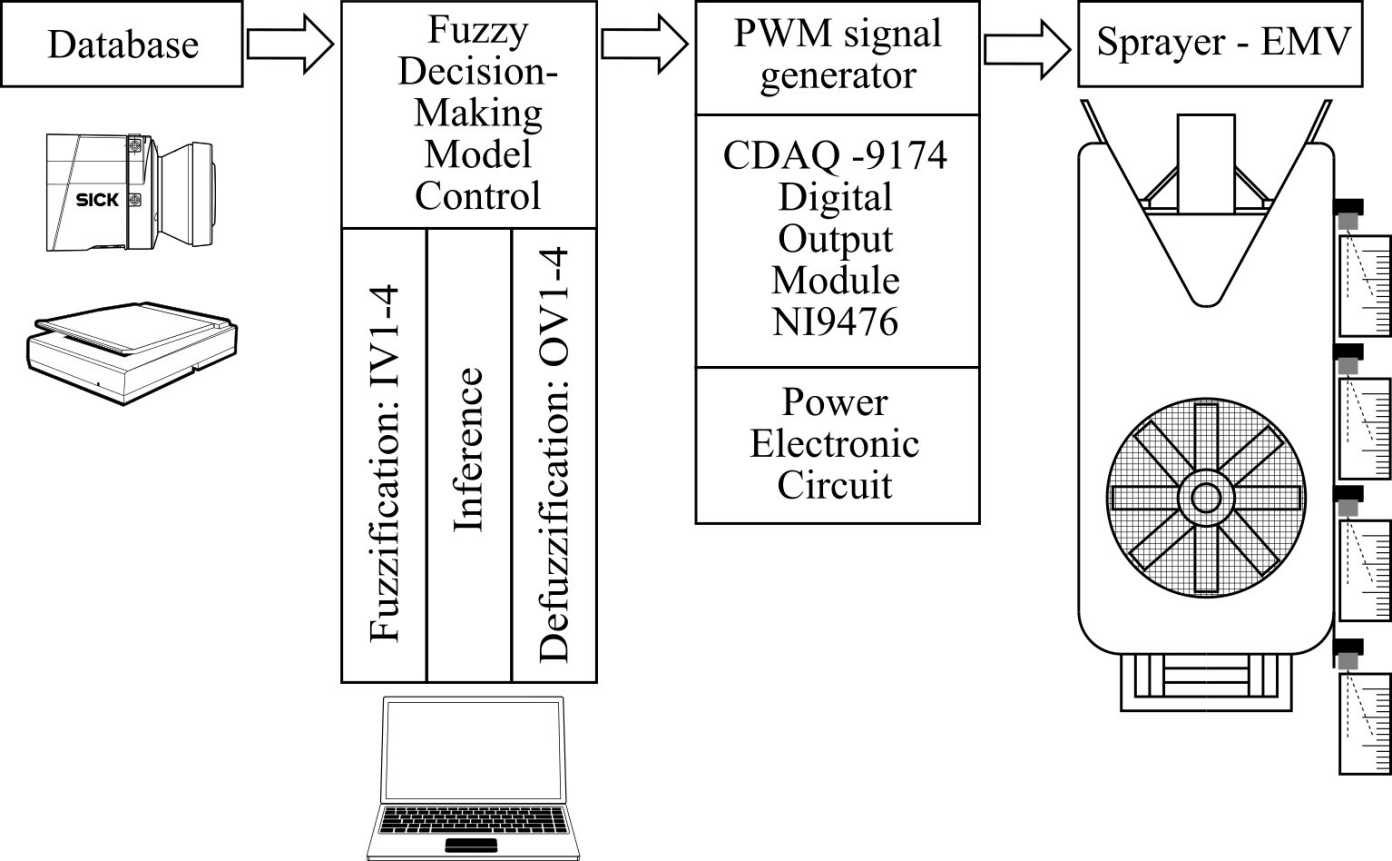
Implementation of the experiment with an automated modular system.

The research work consisted of two main parts. The first part involved determining the tree canopy with the LIDAR measuring system compared to the manually measured leaf area index (LAI) and the number of leaves (Fig 1). The second part included a laboratory trial of spraying with a FLS and is presented in (Fig 2).

#### Orchard

Experiments were carried out in the research orchard (46°30'9.01 '' N, 15° 37'38.94 '' E) owned by the University of Maribor, Faculty of Agriculture and Life Sciences, Slovenia. The size of the orchard surface was 41,000 m^2^. In this intensive orchard plantation, different apple varieties were grafted on a M9 dwarf rootstock, which limits the growth of the above-ground part of the tree (noble varieties), enters earlier into the fruit bearing period and requires a smaller growing crown to achieve the optimum yield. The apple trees were grown in the "narrow spindle" form, which improves the overall volume of trees by intensive cutting. For evaluating the leaf area index, the following varieties of apple trees were randomly selected: (a) 'Golden delicious' (5 years old), (b) 'Jonagold' (7 years old) and (c) 'Gala' (20 years old), at the BBCH91 phenological growth phase, according to recent publication [42].

### Determination of the tree canopy with a LIDAR measuring system and manual counting of leaves and leaf area index

For reconstructing the tree canopy, the density of the leaf was separately measured for the left and right half of the tree with the LIDAR measuring system, which was mounted on a tractor. When the tractor was moving between two rows of trees in the orchard, measurements of distance with LIDAR were carried out on the basis of the laser beam time needed for reflecting the laser light from the left to the right half of the tree. Data was simultaneously recorded in real time on a computer hard drive. The number of points was individually determined from the cloud of points for four individual segments of the left halh and four individual segments of the right half of the tree—altogether 8 segments for each tree (Fig 1, left). The particular values of the number of points in the cloud were then compared to the actual leaf area, which was defined on the basis of manual measurements for each individual segment separately (Fig 1, right). For analysis of the leaf area, 20 trees were randomly selected in the orchard.

LIDAR sensor SICK LMS111 was used, located 140 cm from the ground to the front steel console of the tractor. LIDAR was connected to the Ethernet communication interface with the data storage computer. The SICK LMS111 sensor belongs to the group of active contactless meters and is especially suitable for measuring the natural characteristics of the tree canopy in the orchard. With this sensor, we can measure the distance in the two-dimensional plane in the 270° view sector at a 0.5° resolution. The measurement range of the sensor is from 0.5 m to 20 m at the 50 Hz acquisition frequency and the 12 V DC voltage supply.

Manual measurements of the leaf surface in the experiment were carried out by removing leaves from each individual tree manually from the left and right half of the canopy in the 50 cm width and at heights between 50 to 250 cm. We decided to divide the segments by height, as in (Fig 1), because the spray nozzles are aligned at the same distance. In depth, the segments extended to the middle line of the row represented by the trunk of the tree (Fig 1). The number of leaves and the leaf area index were determined for each individual segment according to the method presented by publication [43].

### FLS for dosage spray mixture control on the principle of a fuzzy logic system

The second part of the research work consisted of the experiment, which included the FLS for dosage spray mixture control. This is shown schematically in (Fig 2). This part of the experiment was carried out under laboratory conditions.

For a FLS (Fig 2 –FLS), we developed an intelligent fuzzy algorithm in the LabVIEW 2015 Fuzzy System Designer (hereinafter, FSD) software package and via a graphic user interface for observing its operation. The FLS was influenced by the width of the duty cycle pulse-width generated signal (Fig 2 - PWM signal generator), which was physically generated at the output of the digital module. After starting an automated dosage control process with a FLS, a dosage of spray mixture (Fig 2 –Sprayer EMV) was measured on each individual segment. For operation, a FLS needs the LIDAR data which were presented in subsection Determination of the tree canopy with a LIDAR measuring system and manual counting of leaves and leaf area index (Fig 2 - Database).

**Fig 3:**
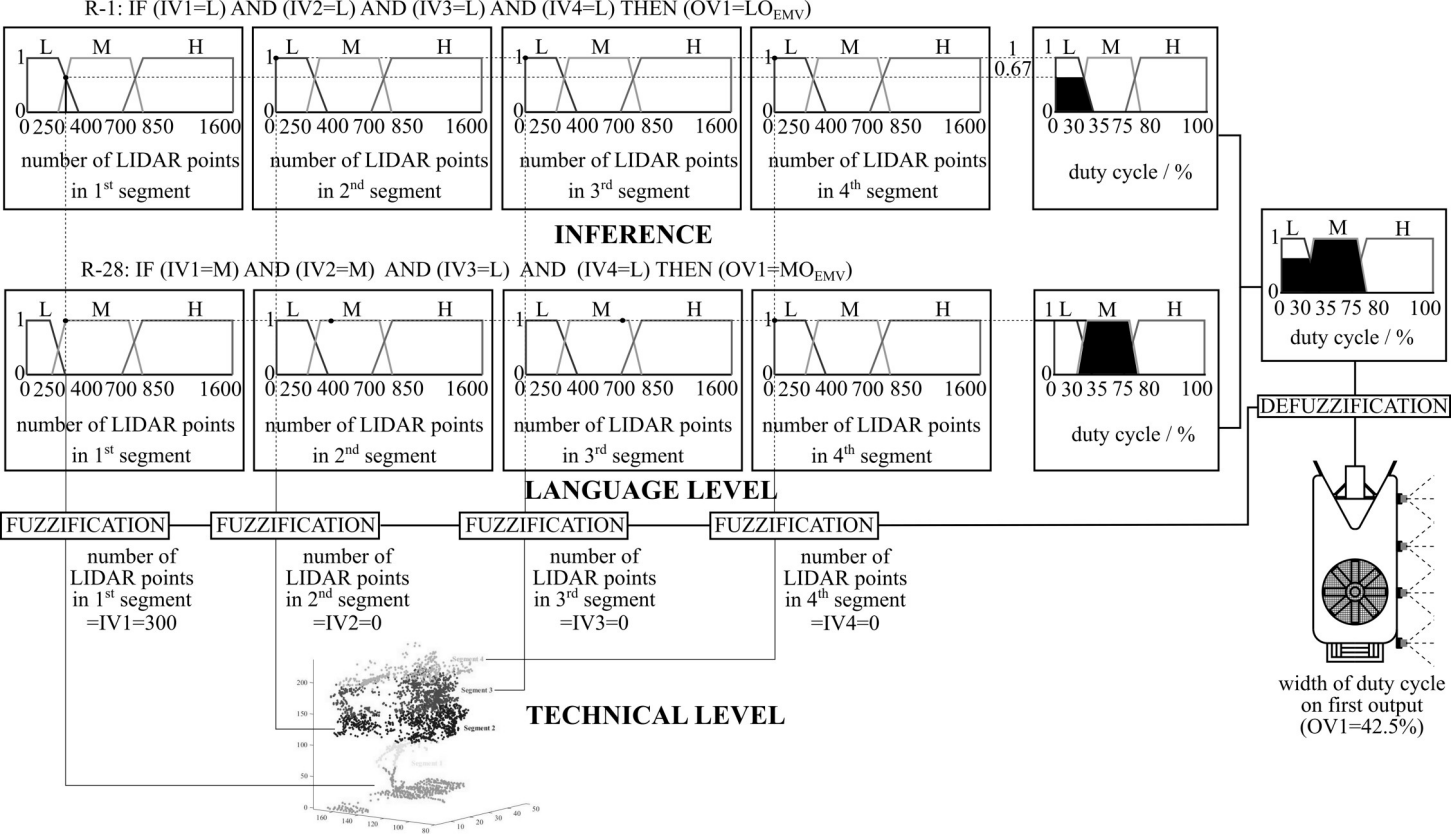
The structure of partial procedure (fuzzification, inference, defuzzification) for dosage control example on a segment, for example, when two rules are valid.

The procedure for designing a FLS based on a fuzzy logic controller consists of three partial procedures: (1) the FUZZIFICATION procedure, (2) the INFERENCE procedure and (3) the DEFUZZIFICATION procedure. All partial procedures were planned using the LabVIEW 2015, FSD software package. All partial implementation of procedures and their sub-processes are illustrated using the example of dosage spray mixture control on an individual tree canopy segment, where the structure of the entire modular automated system for dosage control, as shown in (Fig 3), Fuzzy controller, has four inputs and outputs. These are (a) the input variable IV1 (number of point clouds in the 1st segment), (b) the input variable IV2 (number of point clouds in the 2nd segment), (c) the input variable IV3 (number of point clouds in the 3rd segment), (d) the input variable IV4 (number of point clouds in the 4th segment), (e) the output variable OV1 (variable coefficient on the 1st segment), (f) the output variable OV2 (variable coefficient on the 2nd segment), (g) the output variable OV3 (variable coefficient on the 3rd segment), and (h) the output variable OV4 (variable coefficient on the 4th segment).

#### The fuzzification procedure

The number of input variables (4) was determined by the number of nozzles on the prototype sprayer. We wanted to keep the prototype adequately simple, while at the same time providing good plant protection through sprayer operation flexibility. The first part of the procedure (fuzzification) served to determine the set, interval area and fuzzification of the input variables, named IV1-IV4. First, we determined a set of input and output variables, named OV1-OV4. A set of input and output variables were defined by four inputs (IV1, IV2, IV3 and IV4) and four outputs (OV1, OV2, OV3 and OV4). This means that in controlling dosage on an individual segment of the left and right halves of the tree canopy, at the input of the fuzzy logic controller, four input variables were added, and at the output of the controller four output variables were added (Fig 2). This means that, for each segment of the tree canopy, one input and one output variable are defined. All inputs to and outputs from the fuzzy controller were classified according to the interval areas, which represent those areas where the input (measurement) and output setpoints appear. In our case, the crisp value of the input variables was defined, so that IV1_min_ = IV2_min_ = IV3_min_ = IV4_min_ = 0; IV1_max_ = IV2_max_ = IV3_max_ = IV4_max_ = 1551, where 1551 is the maximum measured number of point clouds with the LIDAR measurement system on an individual segment. Crisp values of output variables were represented by OV1 = OV2 = OV3 = OV4 = 100%, so that OV1_min_ = OV2_min_ = OV3_min_ = OV4_min_ = 0%; OV1_max_ = OV2_max_ = OV3_max_ = OV4_max_ = 100%.

Fuzzification of the input and output variables is determined subjectively, depending on the natural properties of the tree canopy, the impact of disease on the tree canopy in the orchard and the fruit grower’s experience, which control the consumption of the spray mixture dosage, depending on the different tree canopy stages. The tree canopy properties were determined by the number of point clouds on an individual segment, which represents the estimated size of the leaf surface on the individual segment. The estimated values of the leaf surface on the individual tree canopy segment were included in the dosage spray mixture process control. Fuzzification of the crisp input and output variables values was carried out through membership functions (Fig 3), where the trapezoidal form of the affiliation level has a decisive influence on the behavior of the fuzzy controller and represents a basic form of control technology. In our case of dosage control on an individual segment, the following membership functions were defined, which actually represent a discretization level of the FLS:

✓ input membership functions μ_IV1_ to μ_IV4_ and
✓ output membership functions μ_OV1_ to μ_OV4_.

Individual input and output membership functions represent three levels of membership, where, for parametric representation, normalized values of the levels used functions of trapezoidal form with parameters [x1, x2, x3, x4] for input variables (number of point clouds) and parameters [y_1_, y_2_, y_3_, y_4_] for output variables (duty cycle width of pulse-width generated signal). For the example of dosage control on an individual tree canopy segment, the following trapezoid forms of affiliation were defined (written in the form of normalized values), with their terminological descriptions presented in Table 1. We have followed standard procedure according to [44], by doing so we wanted to increase the dosage rate in selected individual tree canopy segments and provide adequate plant protection and yield. Other choices of functions enable higher dosage rate reduction, but the plant protection is questionable.

**Table 1:** Terminological affiliation descriptions for trapezoidal forms which defined the individual input and output membership functions.

Terminological label of affiliation level	Description of terminological affiliation label level	Normalized trapezoidal functions forms with parameters, representing the number of points clouds duty cycle width of pulse-width generated signal in [%]
L_LMS_	Low number of point clouds	x_1_ = 0, x_2_ = 0, x_3_ = 250, x_4_ = 400
M_LMS_	Medium number of point clouds	x_1_ = 250, x_2_ = 300, x_3_ = 800, x_4_ = 850
H_LMS_	High number of point clouds	x_1_ = 700, x_2_ = 850, x_3_ = 1551, x_4_ = 1551
LO_EMV_	Low open EMV	y_1_ = 0%, y_2_ = 0%, y_3_ = 30%, y_4_ = 35%
MO_EMV_	Medium open EMV	y_1_ = 30%, y_2_ = 35%, y_3_ = 75%, y_4_ = 80%
HO_EMV_	High open EMV	y_1_ = 75%, y_2_ = 80%, y_3_ = 100%, y_4_ = 100%

Based on the three levels of membership defining the individual input and output membership functions of the fuzzy controller, it was demonstrated that the parameter in the form of the number of point clouds can partly belong to a set and can form part of two sets.

#### Procedure inference

In the second partial procedure (inference), the composition of a set of rules of control or the composition of the control algorithm were defined and inference operators were formed. A set of control rules forms one of the most important members of the FLS, because with their help, we can connect the input and output variables. A set of control rules was formed from experience (among fruit growers, phytopathologists, leaf area measurement etc.). It is based on good fruit-growing practice, which will be discussed later, along with the individual set rules in Table 2. Based on a set of rules Table 2, the input and output variables were connected; these affect the dynamic behavior of the FLS. The language description of the control system for controlling the dosage of the spray mixture on an individual tree canopy segment was defined as a set of rules using the basic Eq (1), which is written in the following form for the case of Rule 1 from Table 2:

$$if\;(IV1=L_{LMS}\;AND\;IV2=L_{LMS}\;AND\;IV3=L_{LMS}\;AND\;IV4=L_{LMS})\;then\;(OV1={LO}_{EMV}\;AND\;OV2={LO}_{EMV}\;AND\;OV3={LO}_{EMV}\;AND\;OV4={LO}_{EMV})$$ (1)

Other rules are derived in the same way according to Table 2. It includes all possible combinations of input variables by segment—in our example 81 rules. Number of rules is calculated as: number of input affiliation label levels^number of input variables^ = 81. For each such rule, we have decided to create one set of output variables (OV1-OV4).

**Table 2:** Number of rules that influence spray mixture process control.

Rule	Input variables				Output variables			
R	IV1	IV2	IV3	IV4	OV1	OV 2	OV 3	OV 4
1	L_LMS_	L_LMS_	L_LMS_	L_LMS_	LO_EMV_	LO_EMV_	LO_EMV_	LO_EMV_
2	L_LMS_	L_LMS_	L_LMS_	M_LMS_	LO_EMV_	LO_EMV_	LO_EMV_	MO_EMV_
3	L_LMS_	L_LMS_	L_LMS_	H_LMS_	LO_EMV_	LO_EMV_	MO_EMV_	HO_EMV_
4	L_LMS_	L_LMS_	M_LMS_	L_LMS_	LO_EMV_	LO_EMV_	MO_EMV_	MO_EMV_
5	L_LMS_	L_LMS_	H_LMS_	L_LMS_	LO_EMV_	MO_EMV_	HO_EMV_	MO_EMV_
6	L_LMS_	M_LMS_	L_LMS_	L_LMS_	MO_EMV_	MO_EMV_	LO_EMV_	LO_EMV_
7	L_LMS_	H_LMS_	L_LMS_	L_LMS_	MO_EMV_	HO_EMV_	MO_EMV_	LO_EMV_
8	L_LMS_	L_LMS_	M_LMS_	M_LMS_	LO_EMV_	LO_EMV_	MO_EMV_	MO_EMV_
9	L_LMS_	L_LMS_	H_LMS_	H_LMS_	LO_EMV_	MO_EMV_	HO_EMV_	HO_EMV_
10	L_LMS_	L_LMS_	M_LMS_	H_LMS_	LO_EMV_	LO_EMV_	MO_EMV_	HO_EMV_
11	L_LMS_	L_LMS_	H_LMS_	M_LMS_	LO_EMV_	MO_EMV_	HO_EMV_	MO_EMV_
12	L_LMS_	M_LMS_	M_LMS_	M_LMS_	MO_EMV_	MO_EMV_	MO_EMV_	MO_EMV_
13	L_LMS_	H_LMS_	H_LMS_	H_LMS_	MO_EMV_	HO_EMV_	HO_EMV_	HO_EMV_
14	L_LMS_	M_LMS_	M_LMS_	L_LMS_	MO_EMV_	MO_EMV_	MO_EMV_	MO_EMV_
15	L_LMS_	H_LMS_	H_LMS_	L_LMS_	MO_EMV_	HO_EMV_	HO_EMV_	MO_EMV_
16	L_LMS_	M_LMS_	L_LMS_	M_LMS_	MO_EMV_	MO_EMV_	MO_EMV_	MO_EMV_
17	L_LMS_	M_LMS_	L_LMS_	H_LMS_	MO_EMV_	MO_EMV_	MO_EMV_	HO_EMV_
18	L_LMS_	H_LMS_	L_LMS_	H_LMS_	MO_EMV_	HO_EMV_	MO_EMV_	HO_EMV_
19	L_LMS_	H_LMS_	H_LMS_	M_LMS_	MO_EMV_	HO_EMV_	HO_EMV_	MO_EMV_
20	L_LMS_	M_LMS_	M_LMS_	H_LMS_	MO_EMV_	MO_EMV_	MO_EMV_	HO_EMV_
21	L_LMS_	M_LMS_	H_LMS_	L_LMS_	MO_EMV_	MO_EMV_	HO_EMV_	MO_EMV_
22	L_LMS_	H_LMS_	M_LMS_	L_LMS_	MO_EMV_	HO_EMV_	MO_EMV_	MO_EMV_
23	L_LMS_	H_LMS_	L_LMS_	M_LMS_	MO_EMV_	HO_EMV_	MO_EMV_	MO_EMV_
24	L_LMS_	H_LMS_	M_LMS_	M_LMS_	MO_EMV_	HO_EMV_	MO_EMV_	MO_EMV_
25	L_LMS_	M_LMS_	H_LMS_	H_LMS_	MO_EMV_	MO_EMV_	HO_EMV_	HO_EMV_
26	L_LMS_	M_LMS_	H_LMS_	M_LMS_	MO_EMV_	MO_EMV_	HO_EMV_	MO_EMV_
27	L_LMS_	H_LMS_	M_LMS_	H_LMS_	MO_EMV_	HO_EMV_	MO_EMV_	HO_EMV_
28	M_LMS_	L_LMS_	L_LMS_	L_LMS_	MO_EMV_	LO_EMV_	LO_EMV_	LO_EMV_
29	M_LMS_	L_LMS_	L_LMS_	M_LMS_	MO_EMV_	LO_EMV_	LO_EMV_	MO_EMV_
30	M_LMS_	L_LMS_	L_LMS_	H_LMS_	MO_EMV_	LO_EMV_	MO_EMV_	HO_EMV_
31	M_LMS_	L_LMS_	M_LMS_	L_LMS_	MO_EMV_	MO_EMV_	MO_EMV_	MO_EMV_
32	M_LMS_	L_LMS_	H_LMS_	L_LMS_	MO_EMV_	MO_EMV_	HO_EMV_	MO_EMV_
33	M_LMS_	M_LMS_	L_LMS_	L_LMS_	MO_EMV_	MO_EMV_	LO_EMV_	LO_EMV_
34	M_LMS_	H_LMS_	L_LMS_	L_LMS_	MO_EMV_	HO_EMV_	MO_EMV_	LO_EMV_
35	M_LMS_	L_LMS_	M_LMS_	M_LMS_	MO_EMV_	MO_EMV_	MO_EMV_	MO_EMV_
36	M_LMS_	L_LMS_	H_LMS_	H_LMS_	MO_EMV_	MO_EMV_	HO_EMV_	HO_EMV_
37	M_LMS_	L_LMS_	M_LMS_	H_LMS_	MO_EMV_	MO_EMV_	MO_EMV_	HO_EMV_
38	M_LMS_	L_LMS_	H_LMS_	M_LMS_	MO_EMV_	MO_EMV_	HO_EMV_	MO_EMV_
39	M_LMS_	M_LMS_	M_LMS_	M_LMS_	MO_EMV_	MO_EMV_	MO_EMV_	MO_EMV_
40	M_LMS_	H_LMS_	H_LMS_	H_LMS_	MO_EMV_	HO_EMV_	HO_EMV_	HO_EMV_
41	M_LMS_	M_LMS_	M_LMS_	L_LMS_	MO_EMV_	MO_EMV_	MO_EMV_	MO_EMV_
42	M_LMS_	H_LMS_	H_LMS_	L_LMS_	MO_EMV_	HO_EMV_	HO_EMV_	MO_EMV_
43	M_LMS_	M_LMS_	L_LMS_	M_LMS_	MO_EMV_	MO_EMV_	MO_EMV_	MO_EMV_
44	M_LMS_	M_LMS_	L_LMS_	H_LMS_	MO_EMV_	MO_EMV_	MO_EMV_	HO_EMV_
45	M_LMS_	H_LMS_	L_LMS_	H_LMS_	MO_EMV_	HO_EMV_	MO_EMV_	HO_EMV_
46	M_LMS_	H_LMS_	H_LMS_	M_LMS_	MO_EMV_	HO_EMV_	HO_EMV_	MO_EMV_
47	M_LMS_	M_LMS_	M_LMS_	H_LMS_	MO_EMV_	MO_EMV_	MO_EMV_	HO_EMV_
48	M_LMS_	M_LMS_	H_LMS_	L_LMS_	MO_EMV_	MO_EMV_	HO_EMV_	MO_EMV_
49	M_LMS_	H_LMS_	M_LMS_	L_LMS_	MO_EMV_	HO_EMV_	MO_EMV_	MO_EMV_
50	M_LMS_	H_LMS_	L_LMS_	M_LMS_	MO_EMV_	HO_EMV_	MO_EMV_	MO_EMV_
51	M_LMS_	H_LMS_	M_LMS_	M_LMS_	MO_EMV_	HO_EMV_	MO_EMV_	MO_EMV_
52	M_LMS_	M_LMS_	H_LMS_	H_LMS_	MO_EMV_	MO_EMV_	HO_EMV_	HO_EMV_
53	M_LMS_	M_LMS_	H_LMS_	M_LMS_	MO_EMV_	MO_EMV_	HO_EMV_	MO_EMV_
54	M_LMS_	H_LMS_	M_LMS_	H_LMS_	MO_EMV_	HO_EMV_	MO_EMV_	HO_EMV_
55	H_LMS_	L_LMS_	L_LMS_	L_LMS_	HO_EMV_	MO_EMV_	LO_EMV_	LO_EMV_
56	H_LMS_	L_LMS_	L_LMS_	M_LMS_	HO_EMV_	MO_EMV_	LO_EMV_	MO_EMV_
57	H_LMS_	L_LMS_	L_LMS_	H_LMS_	HO_EMV_	MO_EMV_	MO_EMV_	HO_EMV_
58	H_LMS_	L_LMS_	M_LMS_	L_LMS_	HO_EMV_	MO_EMV_	MO_EMV_	MO_EMV_
59	H_LMS_	L_LMS_	H_LMS_	L_LMS_	HO_EMV_	MO_EMV_	HO_EMV_	MO_EMV_
60	H_LMS_	M_LMS_	L_LMS_	L_LMS_	HO_EMV_	MO_EMV_	LO_EMV_	LO_EMV_
61	H_LMS_	H_LMS_	L_LMS_	L_LMS_	HO_EMV_	HO_EMV_	MO_EMV_	LO_EMV_
62	H_LMS_	L_LMS_	M_LMS_	M_LMS_	HO_EMV_	MO_EMV_	MO_EMV_	MO_EMV_
63	H_LMS_	L_LMS_	H_LMS_	H_LMS_	HO_EMV_	MO_EMV_	HO_EMV_	HO_EMV_
64	H_LMS_	L_LMS_	M_LMS_	H_LMS_	HO_EMV_	MO_EMV_	MO_EMV_	HO_EMV_
65	H_LMS_	L_LMS_	H_LMS_	M_LMS_	HO_EMV_	MO_EMV_	HO_EMV_	MO_EMV_
66	H_LMS_	M_LMS_	M_LMS_	M_LMS_	HO_EMV_	MO_EMV_	MO_EMV_	MO_EMV_
67	H_LMS_	H_LMS_	H_LMS_	H_LMS_	HO_EMV_	HO_EMV_	HO_EMV_	HO_EMV_
68	H_LMS_	M_LMS_	M_LMS_	L_LMS_	HO_EMV_	MO_EMV_	MO_EMV_	MO_EMV_
69	H_LMS_	H_LMS_	H_LMS_	L_LMS_	HO_EMV_	HO_EMV_	HO_EMV_	MO_EMV_
70	H_LMS_	M_LMS_	L_LMS_	M_LMS_	HO_EMV_	MO_EMV_	MO_EMV_	MO_EMV_
71	H_LMS_	M_LMS_	L_LMS_	H_LMS_	HO_EMV_	MO_EMV_	MO_EMV_	HO_EMV_
72	H_LMS_	H_LMS_	L_LMS_	H_LMS_	HO_EMV_	HO_EMV_	MO_EMV_	HO_EMV_
73	H_LMS_	H_LMS_	H_LMS_	M_LMS_	HO_EMV_	HO_EMV_	HO_EMV_	MO_EMV_
74	H_LMS_	M_LMS_	M_LMS_	H_LMS_	HO_EMV_	MO_EMV_	MO_EMV_	HO_EMV_
75	H_LMS_	M_LMS_	H_LMS_	L_LMS_	HO_EMV_	MO_EMV_	HO_EMV_	MO_EMV_
76	H_LMS_	H_LMS_	M_LMS_	L_LMS_	HO_EMV_	HO_EMV_	MO_EMV_	MO_EMV_
77	H_LMS_	H_LMS_	L_LMS_	M_LMS_	HO_EMV_	HO_EMV_	MO_EMV_	MO_EMV_
78	H_LMS_	H_LMS_	M_LMS_	M_LMS_	HO_EMV_	HO_EMV_	MO_EMV_	MO_EMV_
79	H_LMS_	M_LMS_	H_LMS_	H_LMS_	HO_EMV_	MO_EMV_	HO_EMV_	HO_EMV_
80	H_LMS_	M_LMS_	H_LMS_	M_LMS_	HO_EMV_	MO_EMV_	HO_EMV_	MO_EMV_
81	H_LMS_	H_LMS_	M_LMS_	H_LMS_	HO_EMV_	HO_EMV_	MO_EMV_	HO_EMV_

The main goal of the linguistic description of the control system for dosage control on an individual tree canopy segment with the rules *''IF–THEN''* is the realization of optimal dosage control, which the fuzzy input information processed in the output. The following sections describe selected, individual rules that are important for good fruit growing practice (see Table 2, marked in green).

#### Interpretation of individual rules relevant to fruit growing practice

The rule R-1 in Table 2 reflects the state of the plant in the initial growth phase. Since all input variables in the case of R-1 are the same (L_LMS_), it was decided to maintain all output variables the same (LO_EMV_). All cases were treated similarly when all input variables were of the same value, eg. M_LMS_ (rule R-39, Table 2). Then all output variables were assigned the same value, that is MO_EMV_. The following rule R-24 is given for the influence of scab, a disease which can completely destroy the tree canopy. Scab attacks the tree canopy from the bottom upwards. In all cases, when the difference between the two adjacent segments is different for two levels, then a lower level of membership on the lower segment is raised by one level. For the R-24 rule, the output variables OV1 were assigned the value MO_EMV_ and not LO_EMV_.

Further explanation is also needed for examples when the value of the input variables on the top of the tree canopy (upper segment 4) for two levels were lower than in one segment lower (segment 3). Such a case appears, for example, in rule R-75. The lower vertical segment 3 with a large number of leaves hid the laser beam path, which in the upper segment 4 apparently measures a lower value number of point clouds. The LIDAR measurement system is positioned substantially lower than the upper segment 4, while the laser beams always derive from the same point in the LIDAR sensor (LMS111). The phenomenon of leaves obscuring each other and overlapping between the third and fourth tree canopy segment was described in subsection Results of LIDAR measurements and comparison with results of the manually measured leaf area (the minimum correlation coefficient was measured in the fourth segment, R = 0.1536). By following the R-75 rule, insufficient estimated leaf area value on fourth segment does not reduce the dosage in the fourth segment (in subsection Analysis of laboratory measurement results the correlation coefficient of the fourth segment was increased on value R = 0.5837). Neither marginal segment has two adjacent segments (for example segments 1 and 4), so they lack the opportunity to link with adjacent segments. Rule R-18 deals with the case when it is between the two input variables with the values H_LMS_ a segment with a value of input variables M_LMS_. Such growth forms of the tree canopy are not common in practice; therefore, it is assumed that also in this case leaves were overlapping in the segment with the value of the input variable M_LMS_ by the two adjacent segments. In order for the middle segment to be insufficiently protected, in the case of rule R-18 and related rules, an output variable on this segment was assigned a value of MO_EMV_. In a similarly meaningful way, the remaining rules in Table 2 were formulated.

#### Example, when for each segment of the tree canopy, several rules exist

In the previous subsection, we dealt with examples in Table 2 where only one rule was active for each tree canopy segment. According to Table 2, an individual tree canopy segment can have several applicable rules. Overlap occurs when the number of point clouds is between 0 and 400 and between 0 and 250. In the case overlapping for determining the output variables *y* Table 1, the ''AND'' procedure was used along with Mamdani's [45] inference operator, which has been written with a fuzzy relational Eq (2):

$$\mu_{IV1\;IN\;IV2(X)\;IN\;IV3(X)\;IN\;IV4(X)}=\min\left\{\mu_{IV1}(x);\;\mu_{IV2}(x);\;\mu_{IV3}(x);\;\mu_{IV4}(x)\right\}$$ (2)

Based on Mamdani's [45] inference operator, a new membership function was created with a set of rules relating to the output variables. This means that for multiple input variables at the output of the fuzzy controller, there is always a fuzzy output set. Modeling of the design procedure inference for dosage control was formed on the example which had four input variables (Fig 3). The example deals with two rules (R-1 and R-28, marked in green) on the basis of Table 2 and the affiliation of membership functions, as in Table 1. After forming a set of dosage control rules on an individual tree canopy segment in the form of Table 2, the defuzzification procedure was continued.

#### Defuzzification procedure

The rules, which are represented in the inference procedure, deliver a fuzzy set of results at the output of the FLS. These need to be converted to crisp values using the defuzzification procedure. This means that the executive actuator (represented by EMV) cannot supply a fuzzy set for its control, but can control the EMV via a crisp value (a crisp value at the output of the FLS, represents a *y* variable), which affects on the duty cycle width of the pulse-width generated signal. In calculating the crisp value of the *y*_DC_ variable, the gravity method COA (Center of Area) was employed [44]. The advantage of the method is shown in the simple calculating of the crisp value. A crisp output value *y*_DC_ represents the coordinate of the center of gravity and corresponds to the duty cycle of opening the EMV with the equation [44]:

$$y_{DC}=\frac{\int_{y_{min}}^{y_{max}}\mu_{OV}(y)∙ydy}{\int_{y_{min}}^{y_{max}}\mu_{OV}(y)dy}$$ (3)

where yDC is the crisp output value at the output of a FLS (%); ymin is the minimal crisp output value at the output of a FLS (%), in our case the minimum value was 0%; ymax is the maximal crisp output value at the output of a FLS (%), in our case the minimum value was 100%; μOV is the output membership function (interval range between 0 and 1). The interval value of yDC ranges from 0 to 100%. This means that a new function is written for calculating the spray mixture dosage, represented by Eq (4):

$$Q_{S}=y_{DC}∙\left(\frac{NFR∙600}{a∙v}\right)$$ (4)

where *Q*_*S*_ is the spray mixture dosage on an individual segment of left and right halves of the tree canopy (L); *y*_*DC*_ is the crisp output value at the output of a FLS (%); *NFR* is the volume flow rate through the nozzle (Lmin^–1^); *a* is the row spacing in the orchard (m); *v* is the constant sprayer travel speed (ms^–1^); and 600 is the conversion factor between different units.

For an overall calculation of dosage for the tree canopy in an orchard one needs add up all the partial flows by individual segments of the left and right halves of the tree canopy:

$$Q=\sum_{s}Q_{S}$$ (5)

where *Q* is the overall quantitative dosage of spray mixture for the left and right halves of the tree canopy (L); *Q*_*S*_ is the dosage of the spray mixture on the individual segment left and right halves of the tree canopy (L).

### Sprayer prototype

The FLS for controlling the dosage of the spray misture, which operated with the help of a FLS, was placed on a conventional sprayer and tested under laboratory conditions. The FLS was used in the laboratory experiment to control the optimal dosage of spray mixture on four segments of the left and right half of the tree canopy. The sprayer prototype (Fig 2) consisted of several components:

(1) FLS (subsection FLS for dosage spray mixture control on the principle of a fuzzy logic system),

(2) EMV trigger system in pulse width mode and

(3) a conventional axial spray connected to an agricultural tractor.

The AGP 200 sprayer is a modern sprayer, consisting of a supporting frame with a chemically resistant polyethylene reservoir and filling sieve, pump, pressure and flow regulator, suction filter, discharge filter, 3-way valves, mixing nozzle and air blower. The axial fan has a diameter of Ø 585 mm and allows the air flow to be directed during the process of applying the mixture drops and adjusting the air flow. Air flow can be changed by adjusting the fan blade angle. Air velocity at discharge is low, so the canopy is not damaged. We modified the sprayer control system so it allows individual activation of 4 nozzles on the left and 4 nozzles on the right. The prototype can operate in both conventional and automated mode. In the powerless state, we do not allow the spray mixture to flow through the EMV; instead, the mixture flows through the return line back into the tank. When the control EMV is under voltage, the normal flow of the mixture through the nozzles is enabled.

The mixture dosage for four canopy segments was controlled by HYPEX FKM MQ control valves (3/2 NC valve with 3 connectors on the housing, rated output power 7 W). We placed these on the adapted spray arbor. The valves have an input and output connector in the housing and a ventilation connector at the top of the magnet sleeve. Control valves operated in direct mode.

In accordance with (Fig 2), the output from the fuzzy controller is led to an automated controller. The Sprayer prototype has a cDAQ-9174 controller (National Instruments, Austin, TX) with a corresponding 32-channel NI-9476 digital output module. On the controller, four physical digital outputs (digital outputs DO0, DO1, DO2 and DO3) were configured to control the doses of the mixture. Digital outputs controlled the power electronic circuit in the pulse-width mode for triggering the EMV, consisting of RT424012 electromagnetic relays (Schrack, Germany). EMVs were incited with10 Hz frequency which ensures optimum application of the spray mixture drops (droplets with a diameter of 70 to 150 μm are predominant in the jet structure), as found by recent publication [46].

The sprayer is equipped with classic vortex nozzles TR-80015C (Lechler GmbH, Metzingen, Germany) with a hollow cone spray. The basic task of the nozzles is to disperse the liquid flow of the spray mixture into the spray with a certain drop spectrum and direct it to the leaf surface, where the droplets of the mixture are deposited evenly over the leaf surface. The nozzle angle is 80°. For the experiment, the operating pressure was set to 10 bar.

### Laboratory experiment

In the laboratory experiment, we tested the operation of the prototype sprayer in two modes (conventional and automated) of the dose control. In the conventional mode, EMVs were continuously fully open, while in the automated mode the width of the pulse width signal was changed.

The process of automated dose control was carried out in real time by experimenting with the values of the number of points in the cloud, which were measured in four segments of the left and right halves of the canopy as part of the experiment in the orchard. We conducted 160 measurements of dosage quantities Q_S_ and 20 measurements of cumulative dosages Q for each treated tree. The volume of the spray dosages was measured with measuring cylinders attached to each nozzle.

After the measurements were completed, a comparative regression analysis was performed between the measured dose levels and the number of points in the cloud. Conventional and automated modes were compared, and dose savings in [%] were estimated. The laboratory experiment was carried out at the Department of Agricultural Engineering of the Agricultural Institute of Slovenia.

## Results

Using the LIDAR automated system, we captured 20 selected canopies in the orchard. Following LIDAR measurement, we removed all the leaves from these canopies and calculated the total leaf area. For verification of our hypothesis, we will confirm that the model is correctly implemented with respect to the conceptual model, so that it matches specifications and assumptions deemed to be acceptable for the given purpose of application to plant protection. In the FLS, rules from Table 2 were included that mimic good grower praxis in plant protection, as has been discussed in the subsection ‘Interpretation of individual rules relevant to fruit growing practice’. Because of this, we based the verification on established correlation analysis of entire datasets. After that, we compared the LIDAR with the manual results.

In the laboratory experiment, the spray mixture dosages through the nozzles were controlled by a FLS with pulse width mode. We conducted a comparative analysis of the use of the spray mixture in either continuous or automated mode. The analysis was carried out for entire plants and for individual vertical segments.

### Results of LIDAR measurements and comparison with results of the manually measured leaf area

**Fig 4:**
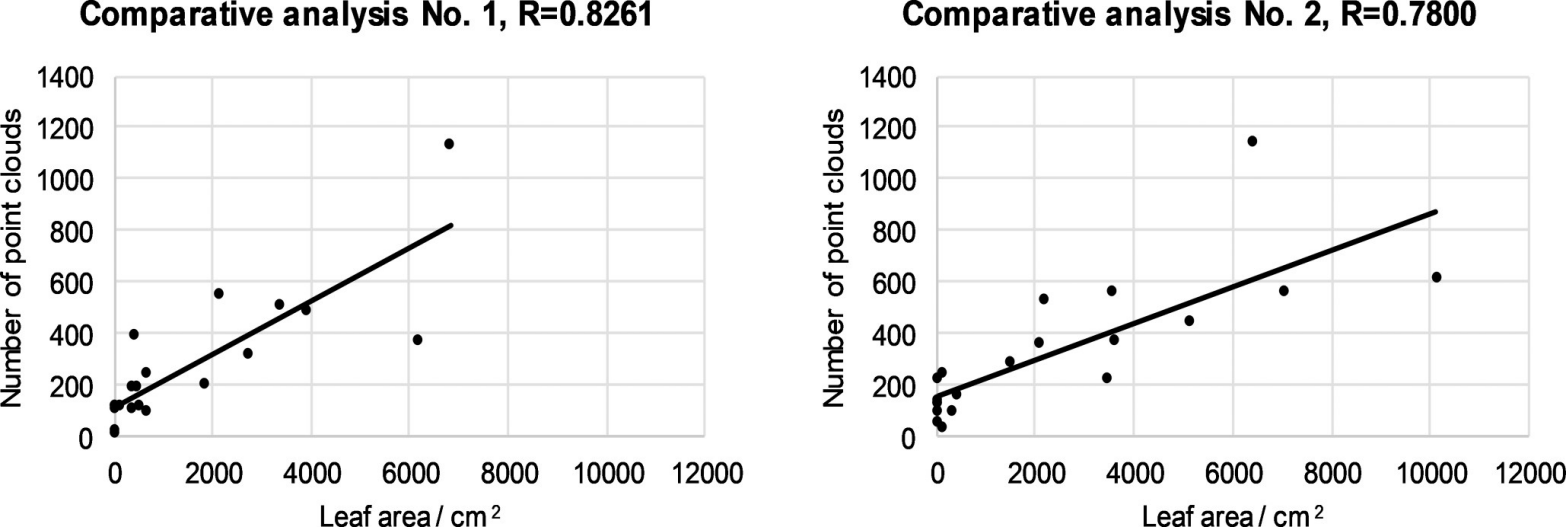
Relationship between number of LIDAR cloud points and leaf area for the first segment left (a) and right (b) half for all 20 canopies.

**Fig 5:**
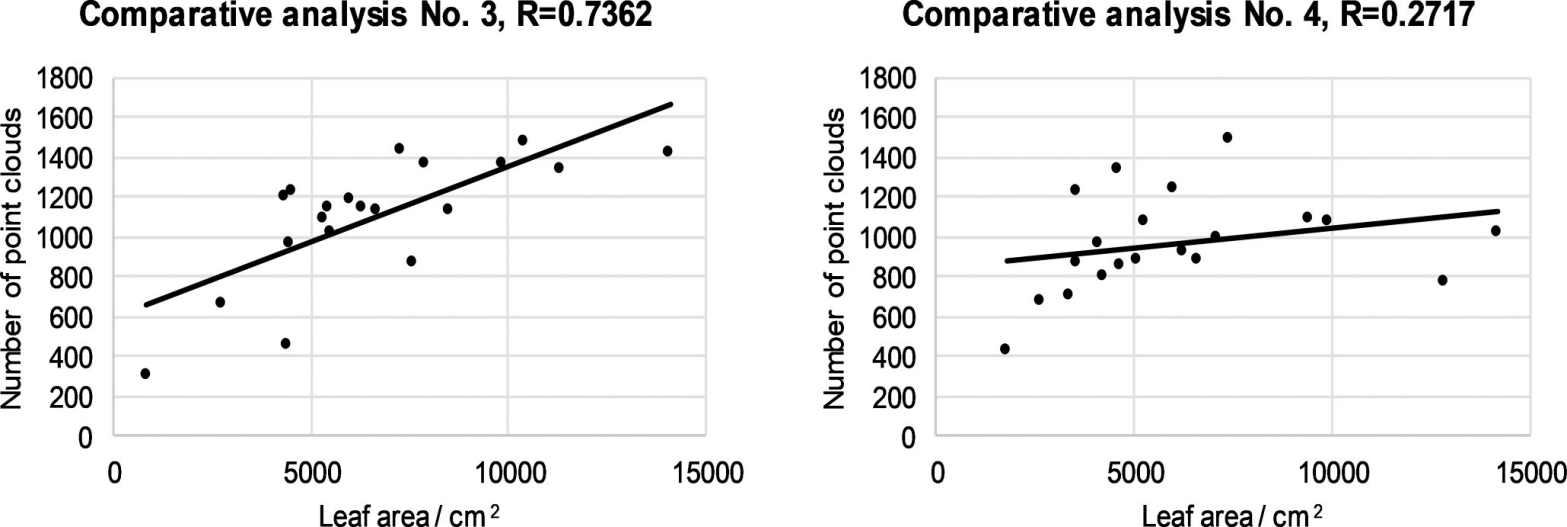
Relationship between number of LIDAR cloud points and leaf area for the second segment left (a) and right (b) half for all 20 canopies.

**Fig 6:**
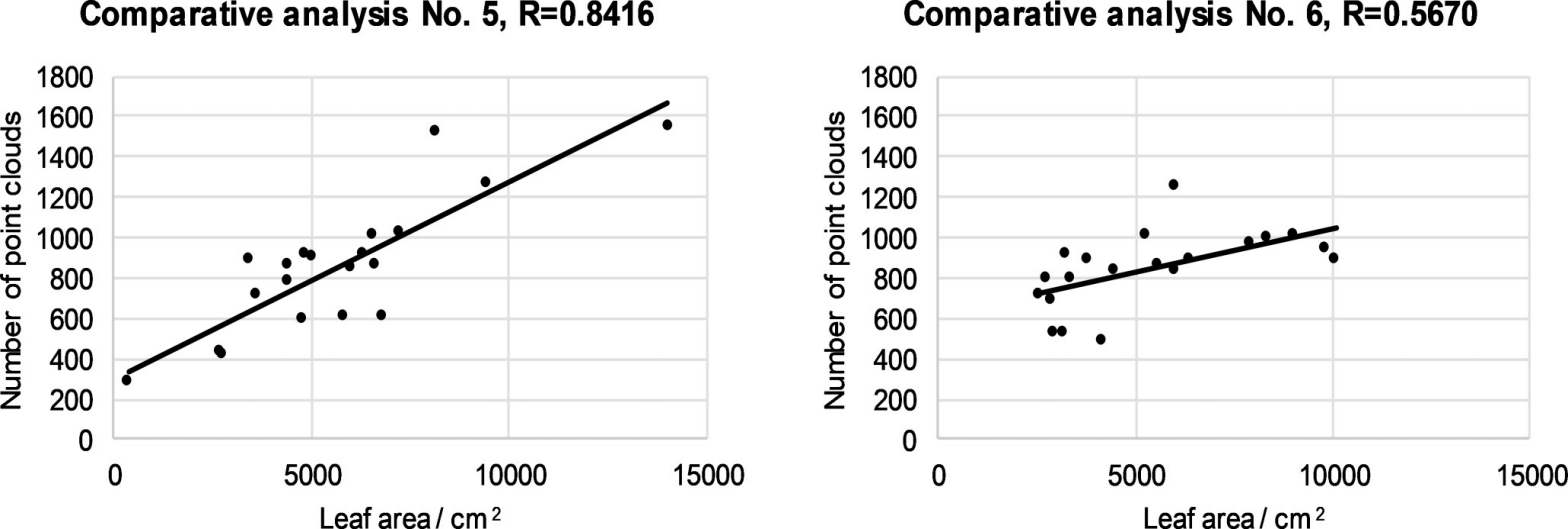
Relationship between number of LIDAR cloud points and leaf area for the third segment left (a) and right (b) half for all 20 canopies.

**Fig 7:**
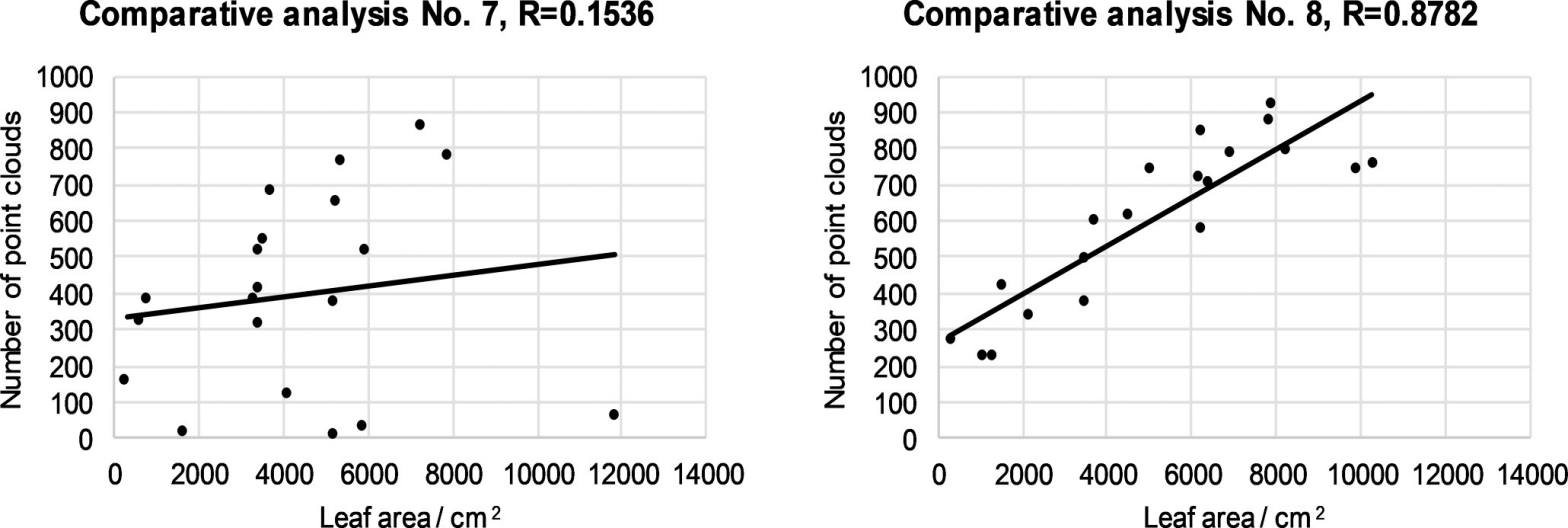
Relationship between number of LIDAR cloud points and leaf area for the fourth segment left (a) and right (b) half for all 20 canopies.

The linear regression method was used to compare the LIDAR and the manually measured leaf area. The results for the first segment are shown in (Fig 4). The results in (Fig 4) are for all 20 canopies. The results are separate for the left and right halves of the canopies. We determined that the linear regression coefficient for the first segment for the left half of the canopies is R = 0.8261 and for the right half of the canopies, R = 0.7808. We analyzed all other canopy segments, the results of which are shown in Figs 5–7.

Depending on the ratio between the number of LIDAR cloud points and the leaf area, it was found that the maximum value of the correlation coefficient was 0.8782 in the case of the fourth segment of the right half of the 20 canopies (Fig 7B). Depending on the ratio of the number of cloud points to the leaf surface, the minimum value of the correlation coefficient is 0.1536 in the case of the fourth segment of the left canopies (Fig 7A). From the value of the correlation coefficient,s we can conclude that in our case there is a positive medium relationship between the two variables.What is exceptional is the value of the correlation coefficient of 0.1536, where there is a slight positive correlation. From the results of the measurements, we can conclude that the automated LIDAR measurement system is comparable to the solutions of other researchers (investigators [47] measured the maximum value of the correlation coefficient of 0.409 with respect to the ratio of the number of reflected laser beams to the leaf area.) and sufficiently effective for the application process controlling spray mixture dosage.

The mean and slight positive correlation between the two variables should be attributed to the fact that we are comparing two different variables and problems in these measurements. Among the problems of measurement were the following: (1) front leaves cover interior leaves near the LIDAR sensor; (2) uneven LIDAR position in the left / right row, owing to the non-ideal movement of the tractor between the rows; (3) the impact of the ruts on the tractor's and LIDAR's slope; (4) the difficulty of detecting the beginning and end of the canopy segments in the direction of travel; (5) changes in the travel speed of the tractor; (6) manual picking of leaves in a particular segment and related problems with determining the edge of the picking zone; (7) the fact that we did not analyze all the leaves in a particular segment but selected them randomly, and (8) environmental conditions such as sunlight, etc.

Difficulties in detecting the number of cloud points with LIDAR depend on the variety, age and growth form of the trees in the plantation. In younger canopies, the leaf density is lower than for older canopies, and consequently the deviation between the two variables in the younger trees is lower.

In particular, we want to highlight the problem in the farthest upper crown segment, where rays from the LIDAR reached at a sharp angle through the third canopy segment. In the event that the third canopy segment is thick, the number of hits in the fourth segment is very low compared to the hand-measured leaf surface. For this reason, we measured the most serious deviation between the measured variables and the lowest value R = 0.1536 in the fourth segment (Fig 7A). Manual measurement, on the other hand, measures all the leaves in the network and is not burdened with this LIDAR problem with respect to the canopy segment coverage.

Other reasons for low corelation between LIDAR and manual measurements are less important. These can be significantly reduced with improved tractor management and the LIDAR tilt control system in the future.

The authors believe that the problem of measuring with LIDAR can be mitigated to a great extent by using a FLS. For example, if the number of LIDAR hits in the third segment is high, a FLS will set a higher dose of spray mixture in the fourth segment than if it had been derived solely from the measured values of the fourth segment. In this article, we have incorporated a correction in the FLS, which increases the dosage amount in any segment, surrounded by two adjacent segments, in which the number of LIDAR hits is high. For example, rule R-18 in the set of rules in Table 2.

In the set of rules in Table 2, we also incorporated corrections that determine the dose of the spray mixture in the case when the third segment has a large number of LIDAR hits. In this case, a FLS increases the dose in the top-level segment for one stage. For example, rule R-4. We acted similarly to the above segments in the case of the lower segments.

Based on the properties of the FLS mentioned above, the authors conclude that the control of pests and diseases will be effective despite lower doses of the spray mixture relative to conventional application. We see this as an advantage over similar methods by other authors.

### Analysis of laboratory measurement results

LIDAR measurements of tree canopies provided a number of cloud points for all canopy segments. The relation to leaf area was discussed above. For the laboratory experiment, measurements of numbers of cloud points enabled dosing control using a FLS and control of spray dosage through the length of the EMV working cycle.

For analysis of laboratory measurements results, a comparative analysis was made between the number of point clouds and the dosage provided to each individual segment for all treated trees. Later, we compared the conventional and automated dosage control processes for evaluation of dose savings.

**Fig 8:**
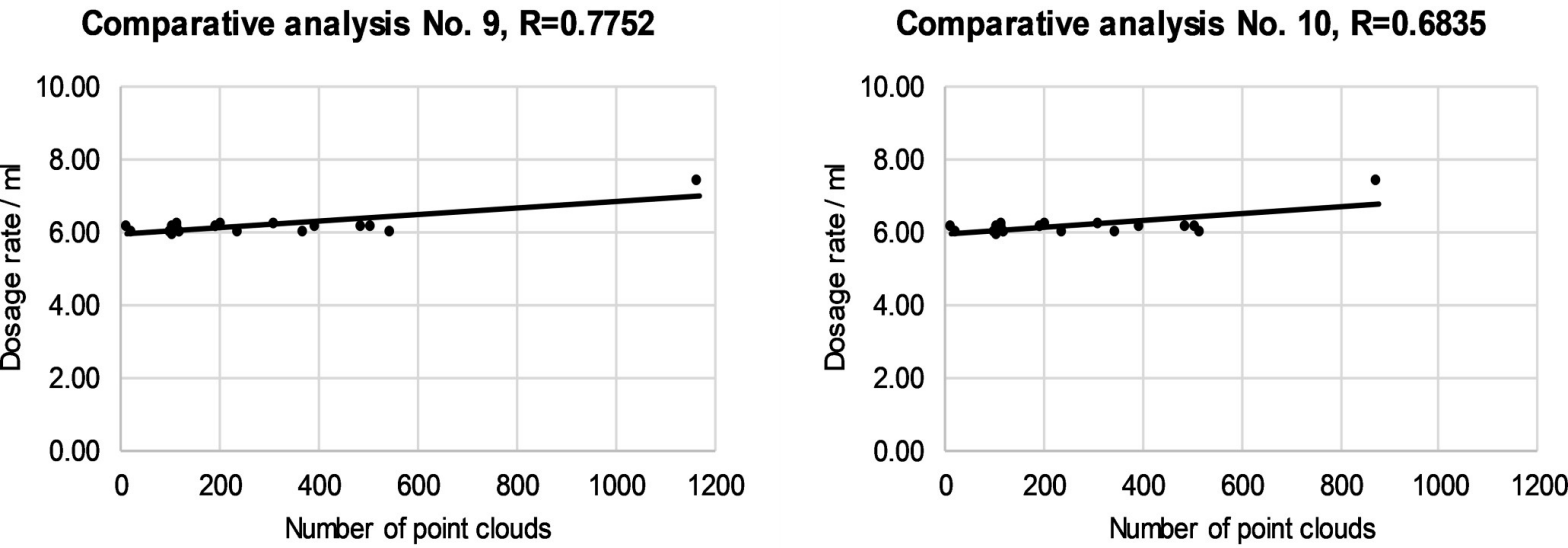
The relationship between the dose and the number of cloud points in the first segment of the left (a) and the right (b) of half of selected canopies.

**Fig 9:**
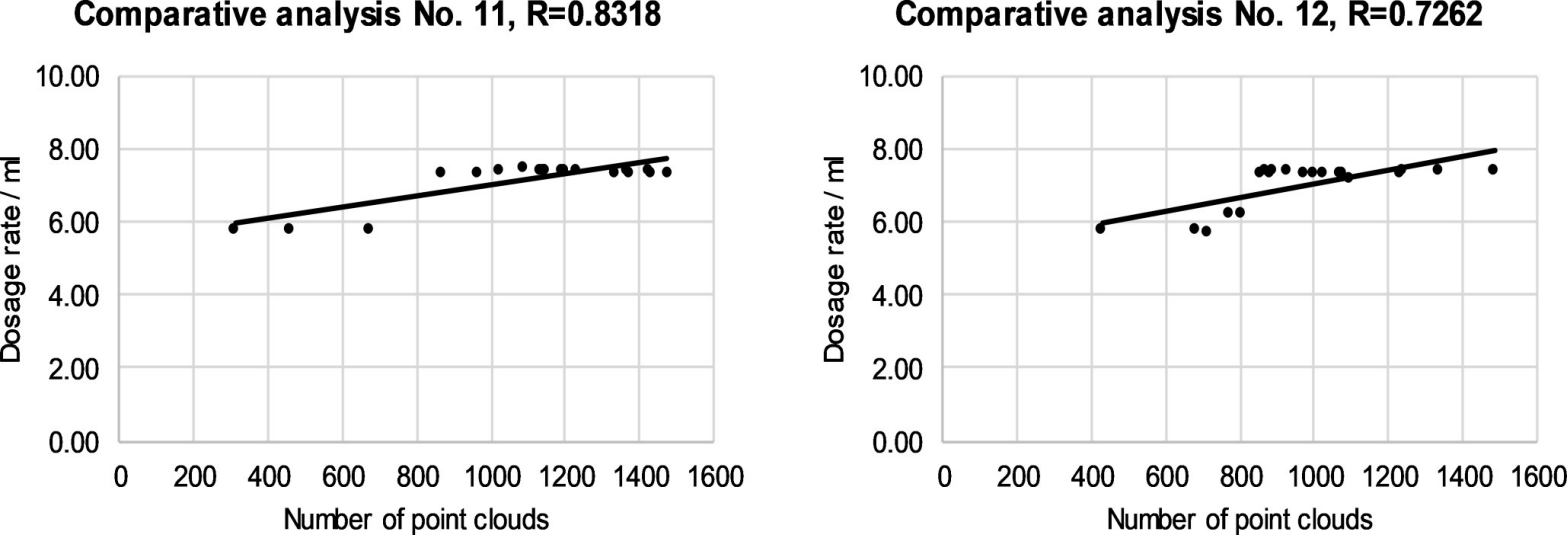
The relationship between the dose and the number of cloud points in the second segment of the left (a) and the right (b) of half of selected canopies.

**Fig 10:**
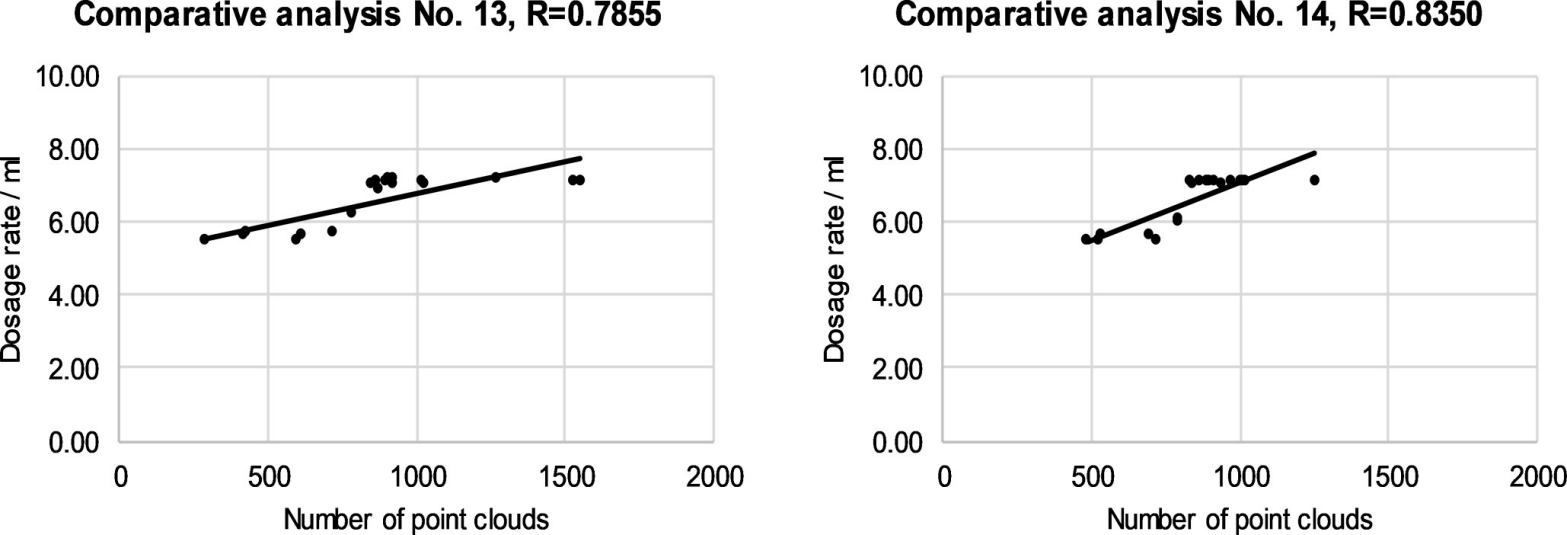
The relationship between the dose and the number of cloud points in the third segment of the left (a) and the right (b) of half of selected canopies.

**Fig 11:**
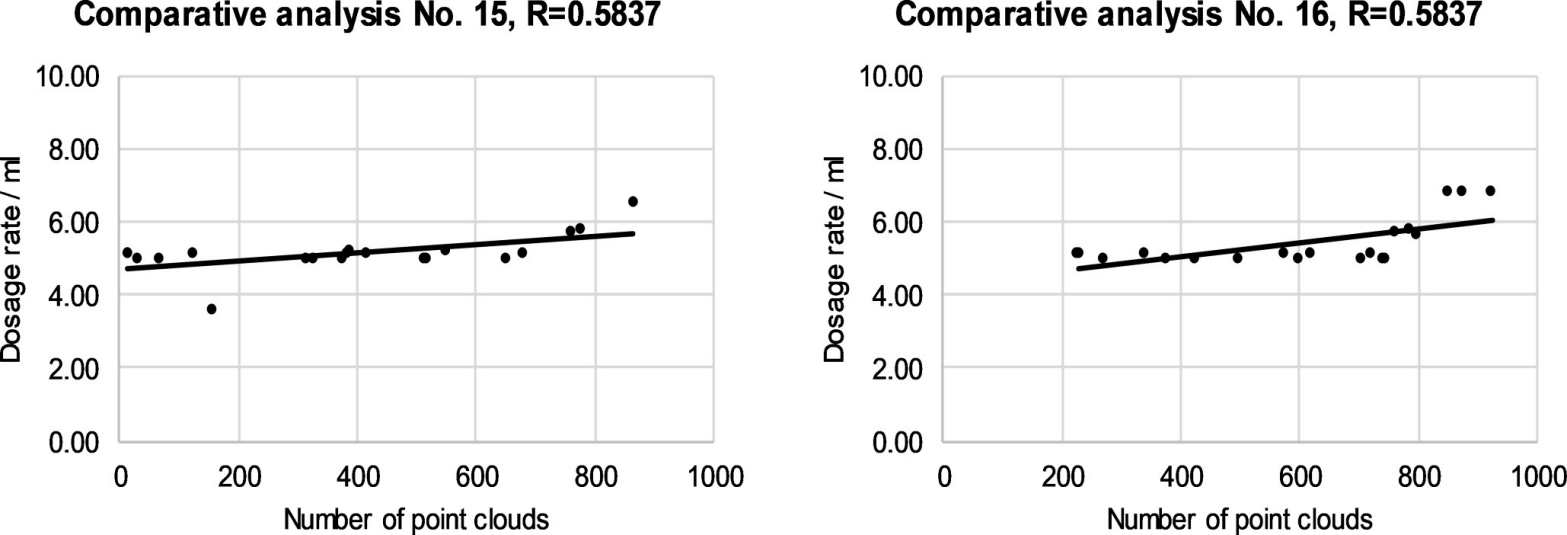
The relationship between the dose and the number of cloud points in the fourth segment of the left (a) and the right (b) of half of selected canopies.

The relationship between the dose and the number of cloud points on individual canopy segments is presented in Figs 8–11. The left and right halves of crowns were evaluated separately. Correlation anaysis between the number of cloud points and the spray dose revealed that the maximum value of the correlation coefficient was 0.8350 in the case of the third segment and right half of canopies (Fig 10B). The minimum value of the correlation coefficient was 0.5837 for the fourth segment of the left half of the canopies (Fig 11A). The calculated values of the correlation coefficients show a positive medium relationship between two variables.

The reasons for the mismatch between the number of cloud points and the leaf area were discussed in the previous subsection. We presented reasons why and how we included individual rules in the FLS and how rules improve the operation of an automated sprayer. In this way, an individual number of cloud points has been assigned different doses, while the relation between the number of cloud points and the dose has become non-linear. The model does not consider individual segments separately from neighboring segments. Figs 8–11 show how an optimized FLS improves the determination of dosages for each segment according to LIDAR-measured tree canopy characteristics with consideration of adjacent segments.

### Dosage savings

To conclude the analysis, we will compare the conventional and automated dosage control processes for all four individual segments of the left and right halves of canopies.

**Fig 12:**
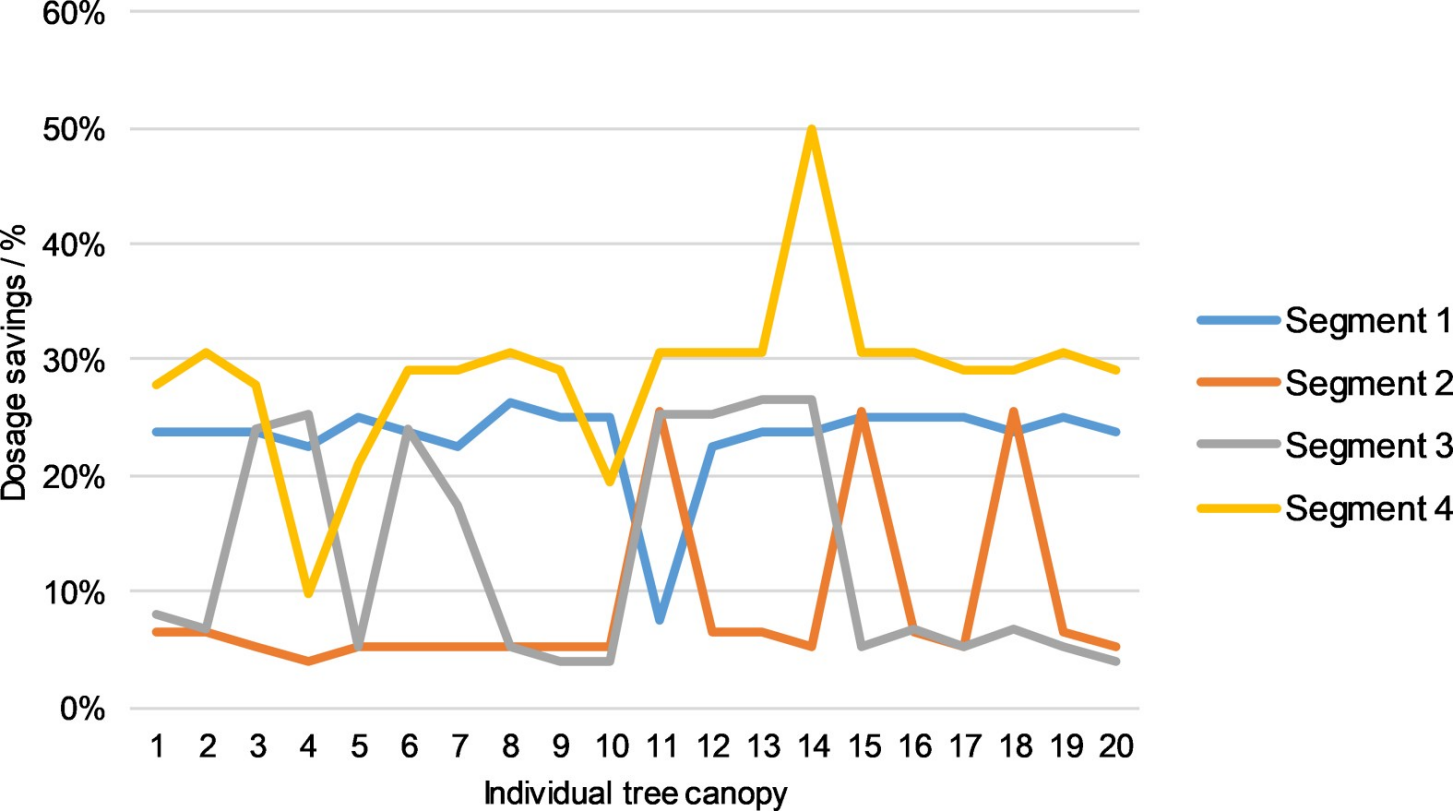
Comparison between conventional and automated dosage control processes on four individual segments of the left half of crowns.

**Fig 13:**
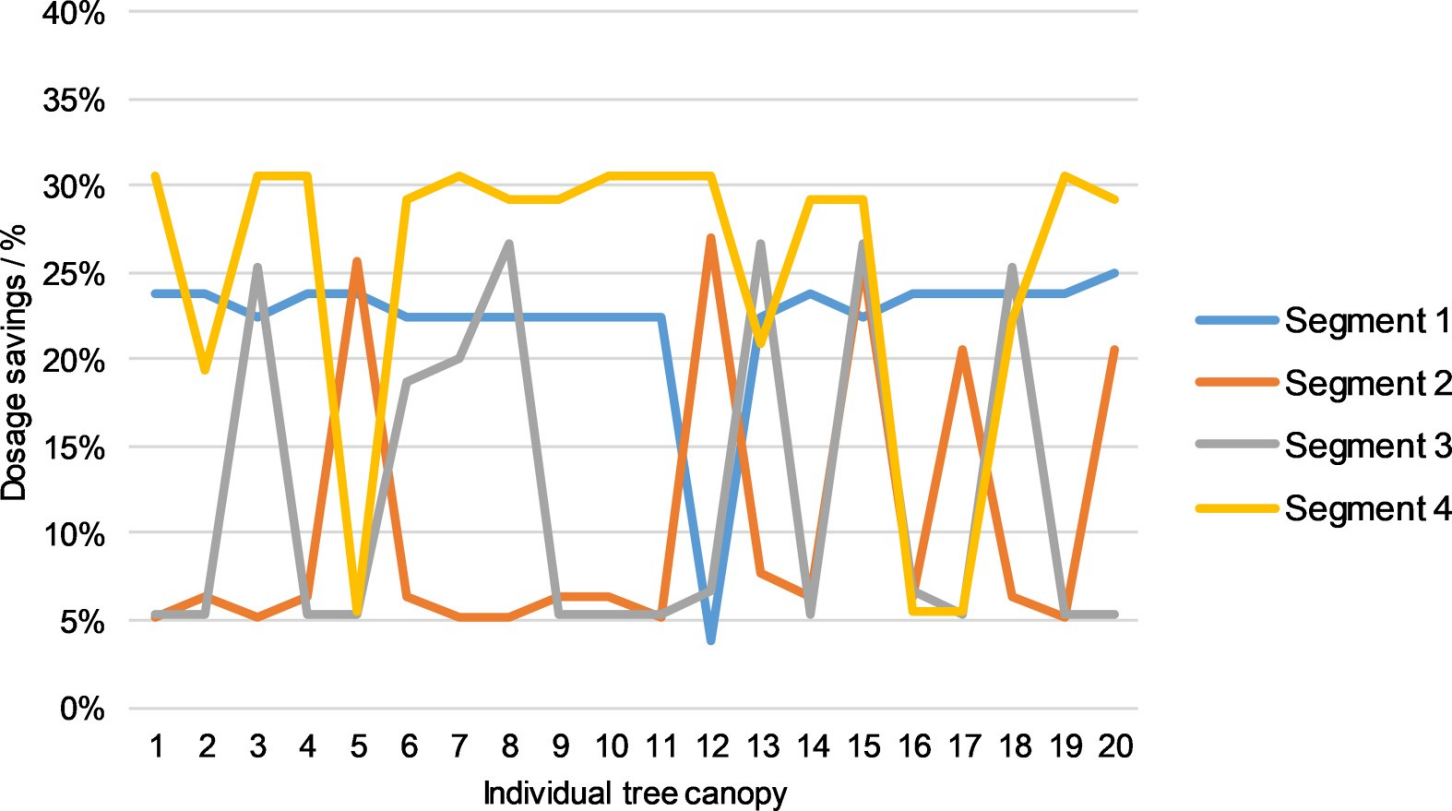
Comparison between conventional and automated dosage control processes on four individual segments of the right half of crowns.

Figs 12 and 13 show dosage savings of the automated dose control procedure with respect to the conventional process. The savings for the left half of canopies for the 1st segment were 23.31%, for the second segment 8.53%, for the third segment 13.07% and for the 4th segment 28.75%. The average spray saving for the left half was 18.41%. For the right half of the canopies, we found that the savings of the spray automated dose control procedure relative to conventional spraying were 22.25% for the 1st segment, 10.45% for the second segment, 12.07% for the third segment and 24.93% for the fourth segment. The average saving on spray for the right half of the 20 analysed canopies was 17.42%. With the comparison of conventional and automated dosage control processes, we seek to draw attention to the excessive consumption of spray in the case of the conventional operational mode of the prototype sprayer. From the results, we conclude that the spray savings in automated mode are significant and amount to 17.92%. This is despite the fact that the trees were in the BBCH 91 phenolic growth phase. We assume that the LIDAR measurement uncertainty for the number of leaves could be significantly lower and the saving in spray doses higher in the case of the phenolic growth phase, e.g. C3 (Mouse ear, individual sheets begin to develop) according to recent publication [42].

From the results of the measurements, we can conclude that the FLS is comparable to the solutions of other researchers. Investigators [48] achieved an average saving on spray from 26% to 27%, while others [13] achieved an average saving of 48%. The authors in [13] and [48] used simple on/off control of the plant protection product dosage rate. This means that the EMV were closed for longer periods, and the dosage of plant protection products was lower. In this paper, we followed good praxis in plant protection: rules from Table 2 were included (for instance, Rule 18, which increase the consumption of plant protection products). In so doing, we also increased the probability of better plant protection and higher yield.

The above results confirm the suitability of the FLS for the spraying of PPP in permanent crops. By reducing the dose of FFS using a FLS, we managed, to the maximum possible extent, to provide environmentally-friendly application, while allowing for the same effectiveness in controlling diseases and PPP with respect to the classic methods of application. This consequently reduces the environmental impact of spraying on the environment.

## Conclusion

In practice, we do not know the uniform standard in dosing quantities of PPP for the same types of permanent crops (orchards) [49]. We therefore compare spray savings with results from other researchers, for instance, from the review paper [49]. It is indeed difficult to compare spray savings in plant protection because (1) the state of the art around the world varies, and (2) we lack reliable reports about the degree of protection when using automated prototype sprayers, which implement various decision-making algorithms. Since the number of drops of PPP spray on the leaf surface in the orcahrd is one of the key factors in the control of diseases and pests, it is assumed that for calculating dosage, the most appropriate application will be based on the measured leaf surface on an individual segment of the tree crown. The process of dosing the PPP was based on the principle of measuring the leaf surface with LIDAR and a FLS that was installed on the prototype of the air blast sprayer. This proves a positive medium relationship between the PPP dose in four individual segments of the left and right halves of the tree crown and the measured number of points in the cloud. Given the ratio between the number of cloud points and the dose, it was found that the maximum value of the correlation coefficient was 0.8350 in the case of the third segment of the right half of the 20-covered tree crowns. The average saving in spray mixture was 18.41% for the left and 17.42% for the right half of the given tree crowns.

The FLS allows the correction of the measured number of points in the cloud of individual tree crown segments. A FLS allows us to include information that builds on the experience of fruit growers and phytopathologists. In this way, we can repair the unfavorable properties of the measurement of leaf surface with LIDAR from one point and ensure adequate protection of the shaded segments, which occur mostly above and below the tree crown. The spraying of the tree crown with a FLS therefore enables the quality protection of all its parts.

The future of the PPP application process is seen in the rapid processing of the properties of tree crowns that will be generated on the basis of powerful LIDAR measuring systems in conjunction with fast-response, non-linear, fuzzy logic control systems, based on regulation that can selectively control the PPP dosage. In this way, we will compensate for the weaknesses of LIDAR measuring systems, such as shading of leaf sheets in the upper part of the plant.

With this control approach, the dosage of the mixture that is selectively applied to the four tree crown segments can be reduced, while at the same time reducing the negative effects on the environment and human beings.

As pointed out in [50] and demonstrated in a series of recent publications (see, e.g., [24–27, 29–31]), user-friendly and publicly accessible web-servers represent the future direction for developing more useful prediction methods and computational tools for practice. In realitly, many practically useful web-servers have already significantly increased the impact of bioinformatics on medical science [51], driving medicinal chemistry towards an unprecedented revolution [52]. For this reason, we shall make efforts in our future work to provide a web-server for the model parameters presented in this paper.
